# Emotional and sensory dysregulation as a possible missing link in attention deficit hyperactivity disorder: A review

**DOI:** 10.3389/fnbeh.2023.1118937

**Published:** 2023-03-02

**Authors:** Anna Grossman, Avi Avital

**Affiliations:** Department of Occupational Therapy, Faculty of Social Welfare and Health Sciences, University of Haifa, Haifa, Israel

**Keywords:** attention deficit hyperactivity disorder (ADHD), emotional dysregulation, sensory dysregulation, developmental stress, methylphenidate (MPH)

## Abstract

Attention Deficit Hyperactivity Disorder (ADHD) is a common developmental disorder affecting 5-7% of adults and children. We surveyed the literature to examine ADHD through three pillars: developmental characteristics, symptomatology, and treatment strategies. Firstly, in terms of developmental characterstics, early life stress may increase the risk of developing ADHD symptoms according to animal models’ research. Secondly, the current core symptoms of ADHD are comprised of inattention, hyperactivity, and impulsivity. However, the up-to-date literature indicates individuals with ADHD experience emotional and sensory dysregulation as well, which early-life stress may also increase the risk of. Finally, we discuss the therapeutic benefits of methylphenidate on both the current core ADHD symptoms and the sensory and emotional dysregulation found in those with ADHD. In summation, we surveyed the recent literature to analyze (i) the potential role of early-life stress in ADHD development, (ii) the involvement of emotional and sensory dysregulation in ADHD symptomatology and finally, (iii) the therapeutic intervention with methylphenidate, aiming to reduce the potential effect of early life stress in ADHD, and mainly emotional and sensory dysregulation. The apparent but currently less recognized additional symptoms of emotional and sensory dysregulation in ADHD call for further investigation of these possible causes and thus increasing treatments efficacy in individuals with ADHD.

## 1. Introduction

Attention Deficit Hyperactivity Disorder (ADHD) is a common developmental disorder suggested to be caused by a delay in brain maturation (Martine Hoogman, 2017), affecting 5-7% of the population (Polanczyk et al., 2007; Thomas et al., 2015; Xu et al., 2018). Risk factors for ADHD development are continuously being investigated, with childhood stress potentially correlating with the development of ADHD itself (Humphreys et al., 2019) and the emotional difficulties often found in children with ADHD (Kennedy et al., 2016). However, due to the difficult nature of controlling for early-life stress in clinical research, there is no definitive clinical evidence for the role of early-life stress on ADHD development.

The core symptomatology of ADHD comprises of inattention, hyperactivity, and impulsivity, thus forming three subtypes of ADHD according to the Diagnostic and Statistical Manual of Mental Disorders (DSM-5) (American Psychiatric Association [APA], 2013). While inattention and/or impulsivity and hyperactivity are the most well-known facets of the disorder, emerging evidence potentially indicates emotional dysregulation (van Stralen, 2016) and sensory processing issues (Sandler, 2001; Panagiotidi et al., 2018) to be potential additional symptoms of ADHD.

Pharmacological strategies for treating ADHD symptoms include methylphenidate (MPH), being one of the most common treatments. This review focuses on MPH specifically since it has a low addictive potential (Robbins, 2002), a shorter half-life than other common ADHD treatments (Manos et al., 1999; Pelham et al., 1999), allowing the comparison of its chronic and acute effects more distinctly, and improving the potential additional symptoms of ADHD: emotional dysregulation (Gamli and Tahiroglu, 2018) and sensory dysregulation (Treister et al., 2015).

Therefore, this review (August 2019 through August 2022) is conducted on three pillars: Developmental characteristics, Symptomatology, and Treatment strategies. Within developmental characteristics, the role of early life stress on the development of ADHD-like symptoms will be discussed. The specific symptoms themselves will then be discussed in the Symptomatology sections, exploring the currently accepted ADHD symptoms (impulsivity, hyperactivity, and inattention), as well as the proposed additional symptoms of ADHD (emotional and sensory dysregulation). Finally, after discussing the way these symptoms develop and the subtleties of the Symptomatology, we will discuss how MPH mitigates ADHD symptoms, including the potential additional ADHD symptoms of sensory and emotional dysregulation. Through this discussion of Developmental Characteristics, Symptomatology, and Treatment strategies, we can follow the line of how early life stress may increase the risk of ADHD symptoms, and how certain ADHD symptoms, particularly emotional and sensory dysregulation, may give rise to the higher order symptoms of impulsivity, hyperactivity, and inattention, and how application of MPH can prevent these symptoms, respectively.

Thus, we aim to understand nuances of the developmental risks, the role of sensory and emotional dysregulation on symptoms, and treatments of ADHD by considering the National Institute of Health’s call to approach a more dimensional diagnostic process for mental health concerns. Furthermore, by understanding the neurophysiological and behavioral symptoms of the disorder and determining the dis-/similarities between ADHD and emotional and sensory dysregulation, we may suggest differential and more accurate treatments of the disorders.

In regards to methodology, we conducted the review between August 2017 through August 2022, as mentioned, through Google Scholar and Pubmed. A limitation worth mentioning is a large subset of these publications were gathered prior to the recent Pubmed website update, meaning some of our citations may not be available through Pubmed currently.

## 2. Developmental characteristics: The Influence of stress on the development of ADHD

Neglect, deprivation, abuse, and trauma may intensify the risk of ADHD development (Humphreys and Zeanah, 2015). The stress of early-life parental separation induced ADHD-like symptoms of hyperactivity and inattentiveness in a rat model, which is then rescued by MPH application (Bock et al., 2017). This correlation between ADHD and early life stress appears also in humans, as the number of stressful life events as evaluated by the Traumatic Events Screen Inventory for children, both in early and later childhood, was associated with higher levels of ADHD symptoms (Humphreys et al., 2019). Furthermore, the rate of ADHD in the U.S. child welfare agencies is 19%, almost 4 times the rate of the general population (Heneghan et al., 2013). Interestingly, children raised for at least 6 months in “depriving” Romanian orphanages similarly have an ADHD rate of 19%, or 4 times the rate of their counterparts raised in “non-depriving” homes (Kennedy et al., 2016) mirroring the increased rate of early life traumatizing institutional deprivation in these Romanian agencies (Rutter et al., 2010). Traumatizing events in later childhood appear to correlate with higher levels of ADHD symptoms, as ADHD symptoms correlate with K-SADS-PL Post-Traumatic Stress Disorder scores in 14- and 15 years-old (Sibley et al., 2020) and sexual and physical abuse before the age of 16 (Vrijsen et al., 2018) or 17 (Singer et al., 2016). Taken together, these results potentially further emphasize the positive correlation found between high Adverse Childhood Experiences report scores and ADHD risk (Brown et al., 2017).

The potential role of early-life trauma on the development of inattention and hyperactivity, symptoms typically associated with ADHD, may be mitigated through environmental factors. Environmental factors like emotionally supportive parenting in childhood and early adolescence correlated with a reduction of emotional dysregulation symptoms in individuals with ADHD (Breaux et al., 2018). Parents of children with ADHD were assessed by the German Family Climate questionnaire at baseline, one year, and two years then compared to offspring’s ADHD symptoms, which indicated that improvement of the family climate (e.g., “In our family everybody cares about each other’s worries”) was associated with a decrease in overall ADHD symptoms (Wüstner et al., 2019). Based on this data, early-life stress occurring along the developmental trajectory correlates with the development of ADHD, but early environmental intervention in both animal and human models may correlate with reduced emotional dysregulation in children with ADHD and the classical ADHD symptoms. While it appears that early life stress may correlate with increased sensory dysfunction, this needs to be further evaluated in those with ADHD specifically. Overall, these data correlate childhood trauma with the development of ADHD; however, further clinical research to determine whether early-life stress has a causative, rather than correlative, effect on ADHD development is required.

## 3. Symptomatology: Emotional and sensory dysregulation as potential ADHD symptoms

### 3.1. Emotional dysregulation in ADHD

Early life stress not only correlates with the typical ADHD symptoms of impulsivity, hyperactivity, and inattention, as above-mentioned, but, according to animal models research, may intensify anxiety-like behaviors in a rat model of “emotional dysregulation” (Ishikawa et al., 2015). Emotional regulation is defined as the “extrinsic and intrinsic processes responsible for monitoring, evaluating, and modifying emotional reactions, especially their intensive and temporal features, to accomplish one’s goals” (Thompson, 1994). Many studies posit emotional dysregulation is a common pathology of ADHD (Retz et al., 2012; Bunford et al., 2018). Indeed, those with ADHD particularly express an elevated anxiety level which subserves as an aspect of emotional dysregulation (Bloch et al., 2017). About 25% of adults with ADHD meet the criteria for generalized anxiety disorder (Piñeiro-Dieguez et al., 2016), and higher anxiety has been associated with greater ADHD symptomatology and even with the most severe form of ADHD (Reimherr et al., 2017).

ADHD has a high comorbidity with a variety of mental disorders, like the previously mentioned generalized anxiety disorder, leading to the exclusion of emotional dysregulation as a direct feature of ADHD, and consider it a manifestation of a comorbid disorder. However, emotional dysregulation has been found in ADHD with no comorbidities, and emotional dysregulation symptoms respond to common ADHD treatments (Reimherr et al., 2005; Corbisiero et al., 2017). Emotional dysregulation was even a diagnostic criterion of ADHD in the DSM II, only to return as an associated feature in the DSM III (Shaw et al., 2014).

Hypothetically, emotional dysregulation may induce some of the classical symptoms of ADHD, like impulsivity and inattention. The dysregulation of emotion would make paying attention more difficult and would also increase the likelihood of impulsive behavior. This hypothesis is mirrored in data of ADHD individuals, as impulsivity and hyperactivity predict the severity of emotional dysregulation in ADHD individuals (Groves et al., 2020). Some studies show that emotional dysregulation is better defined in adult ADHD individuals than children (Retz et al., 2012; Hirsch et al., 2018), but it is still present in children with ADHD (Nigg et al., 2020). Thus, emotional dysregulation remains prominent over the lifespan of those with ADHD (Cubillo et al., 2012), indicating a need for further research on early detection of emotional dysregulation in children with ADHD. Furthermore, treatment options for emotional dysregulation are not specified, and further research is required on how to mitigate emotional dysregulation found in ADHD (Lenzi et al., 2018) as well.

### 3.2. Sensory dysregulation in ADHD

Similar to emotional dysregulation, early life stress correlates with increased sensory dysregulation in adolescents as well (Jeon and Bae, 2022). Both children (Dunn and Bennett, 2002; Ghanizadeh, 2011) and adults (Bijlenga et al., 2017) with ADHD express atypical responses to sensory stimuli. The relation between sensory dysregulation and ADHD is found as early as infancy, as infants with reduced habituation of EEG responses to repeated auditory tones is associated with later attentional dysfunction (Hutchison et al., 2017). This association continues into adulthood, when adults with ADHD were administered questionnaires about sensory processing and ADHD symptoms, the number of sensory difficulties was associated with severity of ADHD (Panagiotidi et al., 2018).

This sensory dysregulation, found in those with ADHD, seems to be tied to emotional dysregulation as well, as pain sensitivity in children with ADHD was recently shown to be associated with the severity of emotional dysregulation (Bruton et al., 2022). This result was corroborated by Lane and Reynolds (2019), as they found that children with ADHD that presented sensory over-responsiveness more often, have clinically significant anxiety levels, and that the strength of the children’s response to sensory stimuli mediates the relationship between attention, anxiety, and sympathetic nervous system recovery (Lane and Reynolds, 2019). Both sensory responsivity and poor reactive control aspect of emotional dysregulation are related to the meso-limbic dopamine system, which has already been associated with the typical ADHD symptoms. Others have related the symptoms of hyperactivity and impulsivity to reactive control and stimulus-driven low level neural responses (Nigg, 2006), and sensory responsivity (Nigg et al., 2005). Some have even argued that heightened sensory sensitivity found in children with ADHD strengthens the connection between hyperactive/impulsive symptoms and emotional lability with three or more clinically impairing ADHD symptoms (DeSerisy et al., 2019).

Neurologically, these disruptions in the processing of somatosensation found in ADHD may be related to disruptions in disruptions in central nervous system inhibitory systems (Parush et al., 2007). The sensory dysregulation found in ADHD seems to share neural correlates between sensory symptoms and intrinsic brain functional connectivity in adults. These neural correlates were also associated with the severity of ADHD symptoms (Itahashi et al., 2020). More specifically, the corpus callosum may play a role in this sensory dysregulation as well, as DTI data from adults with ADHD indicates that the degree of sensory symptoms were parallel to white matter alteration in the large areas of the corpus callosum. Specifically, DTI parameter in the corpus callosum was negatively correlated with sensory sensitivity (Ohta et al., 2020).

## 4. Treatment strategies

### 4.1. Methylphenidate

Methylphenidate (MPH) is one of most commonly prescribed ADHD medications and is highly efficacious in treating ADHD symptoms (Pliszka et al., 2017). MPH prevents the reuptake of norepinephrine and dopamine within their respective neuroregulation systems (Hannestad et al., 2010; Ihezie et al., 2019). Consequently, MPH reduces resting state functional connectivity (RSFCs) in a large network involving cortical regions, cerebellar regions, and the visual, executive, and default mode networks. In adolescents with ADHD, connectivity in these regions may be heightened; MPH may be “normalizing” these ADHD individuals RSFCs (Silk et al., 2017). Chronic administration of MPH does not appear to increase addictive tendencies (Robbins, 2002) likely because MPH does not promote electrophysiological dopamine signal bursts like with other illicit stimulants (Swanson and Volkow, 2002). MPH does not appear to cause long-term consequences on learning, memory, or social behavior (Martin et al., 2018), and its effects are reversible (Silk et al., 2017).

#### 4.1.1. Acute administration of methylphenidate

Both acute and chronic MPH administration increases activity in the Ventral Tegmental Area (VTA) and Locus Coeruleus (LC) (Karim et al., 2017). Acute MPH application normalizes striatal dopamine levels (Aarts et al., 2015) and has age-dependent effects (Schrantee et al., 2017). Acute MPH appears to have widespread effects on neural connectivity. The acute administration of the drug alters both LC and VTA/Substantia Nigra Pars Compacta connectivity between several brain regions (Kline et al., 2016). However, acute MPH application has a dose-dependent effects on LC firing, with low dose suppressing LC discharge (Devilbiss and Berridge, 2006), while typical dosing increasing LC firing rate in rats (Tang and Dafny, 2012). Overall, acute MPH application may have age- and dose-dependent effects on the dopaminergic and noradrenergic systems, which should be accounted for when determining the most effective therapeutic dosage.

Acute MPH has a higher efficacy in treating attentional symptoms than impulsive symptoms (Dougherty et al., 2016). Through the improvement of attention, acute MPH improves cognitive performance even in healthy individuals (Schmidt et al., 2017), perhaps by reducing noradrenergic and dopaminergic dysfunction (Howlett et al., 2017). Acute MPH may rescue this noradrenergic and dopaminergic dysfunction through enhancing the transport of amino acids involved in NE and DA metabolism (Quansah et al., 2017a). Acute MPH application normalizes pupil size, a correlate of the LC-NE system, during attentional performance in children with ADHD (Wainstein et al., 2017). During reward sustained attention tasks, acute administration of MPH regulated the increased activation in the orbitofrontal cortex in children with ADHD (Rubia et al., 2009). Furthermore, acute administration of MPH has been found to reduce right amygdala reactivity in both children and adults with ADHD, but reduces left amygdala reactivity only in adults. These age-dependent effects of acute MPH on amygdala reactivity are important to note, as amygdala reactivity is one of the functional neural mechanisms underlying emotional processing (Bottelier et al., 2017). As noted above, the amygdala changes significantly throughout development, which may cause age-dependent effects of the medication on amygdalar functioning. Acute doses of MPH appear to be anxiolytic (Segev et al., 2016; Lelieveld et al., 2019), and alter reactions to fear stimuli (Ritov and Richter-Levin, 2017). Many studies find MPH induces locomotion, which suggest the drug is anxiogenic, but this heightened locomotion is likely independent of anxiety-like behaviors (Boyette-Davis et al., 2018); in humans, enhanced somatomotor functional connectivity to the thalamus and striatum, is increasing motor function (Farr et al., 2014), offering a potential mechanism in which MPH increases motor function without an increase in anxiety. Nonetheless, the anxiolytic mechanism of MPH is up for debate. MPH improves cognition, which may allow for anxiety to be processed cognitively, rather than the drug being directly anxiolytic (Ernst et al., 2016). Interestingly, acute MPH application may exert anti-nociceptive properties on adults with ADHD, who had increased sensitivity to pain compared to controls, indicating a potential therapeutic relation between MPH and sensory dysregulation found in ADHD as well (Treister et al., 2015). Ultimately, even acute doses of MPH reduce anxiety, a component of emotional dysregulation, and appear to stabilize some aspects of sensory dysregulation, potentially relating to acute MPH application as a therapeutic treatment of some aspects of sensory and emotional dysregulation found in ADHD. A summarization comparing the effects of acute and chronic MPH application has been supplied in Table 1.

**TABLE 1:** A comparison of the effects of acute or chronic methylphenidate administration.

	Acute MPH administration	Chronic MPH administration
**Physiological**		
Increase in LC activity	Healthy adults 45 mg single administration (Kline et al., 2016)	Rats 2.5 mg/kg, i.p 6 days (Tang and Dafny, 2012)
Increase in VTA activity	Rats 0.6, 2.5, and 10.0 mg/kg, i.p single administration. (Kharas et al., 2017)	Rats 0.6, 2.5, and 10.0 mg/kg, i.p 6 days. (Kharas et al., 2017)
Increase in trophic factors	-	ADHD and controls children 18 mg up to 54 mg 4 weeks (Akay et al., 2018)
Reduces inflammation	-	Rats 2.7 mg kg, i.p 5 weeks (Aga-Mizrachi et al., 2014)
Age-dependent affects	ADHD and controls children and adults 0.5 mg/kg single administration. (Bottelier et al., 2017)	ADHD children and adolescents unknown dose 3 months (Wolff et al., 2016)
Sensory dysregulation	ADHD and controls adults average dose 14.2 ± 6.3 mg single oral dose. (Treister et al., 2015)	
**Behavioral**		
Increase in cognition	Healthy adults 60 mg single oral dose. (Schmidt et al., 2017)	Rats 5 mg/kg 19 days (Leary et al., 2017)
Reduces anxiety	Healthy subjects 20 mg single oral dose. (Segev et al., 2016)	Mice 3 mg/kg 16 days (Deslauriers et al., 2019) ADHD children and adolescents increase doses from 5 mg to 40 mg 8 weeks (Snircova et al., 2016)
Reduces emotional dysregulation	–	ADHD adolescents unknown dose 6 months (Gamli and Tahiroglu, 2018) ADHD children 5-mg/d escalations up to 60 mg/d 12 months. (Kutlu et al., 2017)

MPH, methylphenidate; LC, locus coeruleus; VTA, ventral tegmental area.

#### 4.1.2. Chronic administration of methylphenidate

Chronic MPH administration increases neuroplasticity in a variety of brain regions (Simchon-Tenenbaum et al., 2015; Quansah et al., 2017b) and upregulates expression of genes associated with plasticity and dendritic spine formation (Quansah et al., 2017b). MPH has been shown to increase trophic factors, specifically BDNF serum levels in both ADHD individuals and healthy controls (Akay et al., 2018), which may be responsible for the increase in neuroplasticity and cognition. Chronic MPH application at the typical dose increases LC firing in rats (Tang and Dafny, 2012), which is to be expected, an MPH increases NE release. Dopaminergically, chronic application of MPH increases D1 receptor expression in the striatum in rats, and increases D2 receptor expression in female rats (Izquierdo et al., 2016). More generally, MPH also reverses high levels of inflammation (Aga-Mizrachi et al., 2014), which may indirectly reduce overall ADHD symptoms, as high levels of inflammation are associated with ADHD (Dunn et al., 2019). In an age-dependent manner, chronic MPH application has been shown to increase blood flow within the thalamus and striatum of older children, but not adults (Schrantee et al., 2016). Additionally, the adolescent pFC is more susceptible to neuromodulation from chronic MPH use than adults (Venkataraman et al., 2019) and affects boy’s white matter more than men’s (Bouziane et al., 2019). These age-dependent, neurological effects of MPH mirror the developmental trajectory of ADHD.

After MPH treatment, adolescents with ADHD report improved quality of life (Karci et al., 2018). Regarding sensory dysregulation, pain perception in children and adolescents with ADHD is regulated with chronic MPH application (Wolff et al., 2016). Furthermore, the stimulant lessens anxiety (Snircova et al., 2016; Deslauriers et al., 2019; Jager et al., 2019), and has been shown to decrease emotional dysregulation in children with ADHD (Kutlu et al., 2017; Gamli and Tahiroglu, 2018; Ventura et al., 2022) potentially through reducing impulsivity (Gamli and Tahiroglu, 2018). MPH may lessen anxiety through the reduction of dopaminergic functional connectivity between the amygdala and the rostral anterior cingulate cortex (Berry et al., 2019). Chronic administration of MPH also reported to decrease blood flow to the orbitofrontal cortex in children with ADHD, providing an additional mean through which MPH may reduce anxiety (Lee et al., 2005). While the potential efficacy of MPH on emotional and sensory dysregulation warrants additional investigation, the role of ADHD medications with different mechanisms of action than MPH may elucidate on differing neurobiological mechanisms of these dysregulations as well.

## 5. Conclusion

The current core ADHD symptoms are comprised of inattention, hyperactivity, and impulsivity (American Psychiatric Association [APA], 2013). Despite not being current diagnostic criterion, emotional and sensory dysregulation appear to be key components of ADHD as well (Dunn and Bennett, 2002; Ghanizadeh, 2011; Retz et al., 2012; Bijlenga et al., 2017; Bunford et al., 2018). Table 2 describes potential objective, physiological diagnostic tests for emotional dysregulation, sensory dysregulation, and attentional dysregulation found in ADHD. The pupil reflex test, correlates pupil constriction and subsequent dilation in response to light to attentional dysregulation (Wainstein et al., 2017) and autonomic dysfunction (Hamrakova et al., 2019, 2020; Bellato et al., 2021) in ADHD. Additionally, if we further explore the relation between emotional and sensory dysregulation and the classical ADHD symptoms, more potential diagnostic practices may arise.

**TABLE 2:** Potential physiological diagnostic tests for attention deficit hyperactivity disorder: Studies mentioned evaluated sensory, emotional and attentional dysregulation.

	Subjects	Dose amount	Treatment duration	Results
**Sensory dysregulation**				
Cold pressor test (Treister et al., 2015)	Thirty subjects with ADHD and 30 controls	Individual therapeutic dosages of Methylphenidate (Short acting Ritalin IR)	Single administration	Shorter cold pain threshold was lower among ADHD before treatment and recovered post-treatment.
Electro-dermal activity (Lane and Reynolds, 2019)	Children with ADHD			Partial presentation of sensory over responsivity and negative impact on activity and participation
Electro-dermal activity (McIntosh et al., 1999)	Nineteen children with sensory-modulation disruptions (SMD) and 19 controls			Children with SMD showed more and larger EDR than control children
Pre-pulse inhibition (Holstein et al., 2013)	Twenty-six ADHD patients and 26 controls			Normal PPI
Pre-pulse inhibition (Schulz-Juergensen et al., 2014)	Nineteen children with ADHD with/without MPH treatment	The median regular dosage of MPH was 10 mg (range = 7.5-60 mg)	Single administration	PPI was lower in ADHD compared with controls. MPH had no effect
**Emotional dysregulation**				
Salivary cortisol (Lane et al., 2010)	Thirty-nine children with ADHD and 46 controls			Cortisol level was similar in both groups
Electro-dermal activity (Dolev et al., 2021)	Fifty-seven patients with breast cancer pre-/post-radiotherapy			Electro-dermal activity was similar
Electro-dermal activity (Vernetti et al., 2020)	Forty-one toddlers with ASD and 32 controls			Lower EDA responses to threatening events among ASD
Electro-dermal activity (Airij et al., 2020)	Thirty-five children with ASD and 55 controls			Blunt EDA responses to stressful stimuli among ASD
Electro-dermal activity (Beauchaine et al., 2015)	Ninety-nine preschoolers with ADHD and 41 controls			Electro-dermal activity was similar
Startle response (Dolev et al., 2021)	Fifty-seven patients with breast cancer pre-/post-radiotherapy			Startle response was similar
Heart rate variability (Bunford et al., 2017)	Forty-eight children with ADHD and 56 controls			HRV was similar
Heart rate variability (Griffiths et al., 2017)	229 children and adolescents with ADHD and 244 controls			HRV was similar
**Attentional dysregulation**				
Auditory sustained attention test (ASAT) (Dolev et al., 2021)	Fifty-seven patients with breast cancer pre-/post- complementary alternative medicine (CAM)			CAM ameliorated the radiotherapy-induced decrease in ASAT as observed 1 month post-radiation period
Pre-pulse inhibition (Holstein et al., 2013)	Twenty-six-eight adult with ADHD and 26 controls			PPI was similar
Pre-pulse inhibition (Schulz-Juergensen et al., 2014)	Nineteen children with ADHD with/without MPH treatment	The median regular dosage of MPH was 10 mg (range = 7.5-60 mg)	Single administration	PPI was lower in ADHD compared with controls. MPH had no effect
Pupil reflex test (Wainstein et al., 2017)	Twenty-eight adolescents with ADHD and 22 controls	Regularly prescribed dose		Decreased pupil diameter among ADHD which recovered post-MPH treatment

Emotional and sensory dysregulation may give rise to the current core symptoms of ADHD, which in turn induce higher order, classical symptoms. For instance, dysregulated emotions and improperly processed sensory input is quite distracting of itself. Sensorimotor gating is a preconscious regulator of attention, and is why the pre-pulse inhibition of startle is often impaired in disorders with attentional abnormalities (Feifel et al., 2009), and visuospatial working memory may underlie choice-impulsivity in ADHD (Patros et al., 2015). Emotional and sensory dysregulation can induce higher emotionality which in turn can increase impulsivity. Research supports this hypothesis, as higher levels of sensory issues in ADHD can predict aggression levels (Mangeot et al., 2001), impulsivity and hyperactivity can predict the severity of emotional dysregulation in ADHD (Groves et al., 2020), and heightened sensory sensitivity strengthens the relationship between hyperactive/impulsive symptoms and emotional dysregulation in children with three or more clinically impairing ADHD symptoms (DeSerisy et al., 2019). Therefore, we suggest that emotional and sensory dysregulation give rise to the core classical symptoms of ADHD of impulsivity, inattention, and hyperactivity.

On the treatment end, we conclude that MPH mitigates ADHD symptoms once they have developed. Acute and chronic MPH administration has beneficial effects on ADHD symptomatology (Dougherty et al., 2016; Pliszka et al., 2017; Karci et al., 2018). Acute and chronic administration of MPH in those with ADHD reduces sensory dysregulation (Durukan et al., 2011; Treister et al., 2015; Shang et al., 2020), emotional dysregulation (Segev et al., 2016; Kutlu et al., 2017; Lelieveld et al., 2019; Ventura et al., 2020). Hypothetically, as above-discussed, MPH treatment effectiveness on the ‘classical’ ADHD symptoms may be due to its’ additional role in lessening sensory and emotional dysregulation, preventing the development of higher-order symptoms of impulsivity, hyperactivity, and inattention. Overall, MPH proves to be an appropriate, relatively safe therapy for ADHD, normalizing the neurological and behavioral deficits found in the disorder. Further clinical measures may be taken in both the evaluation and treatment of ADHD, with an integration of sensory and emotional dysregulation into the diagnostic process and the application of psychotherapies targeting these dysregulations a well in populations with ADHD.

These results lead us to conclude that: (i) developmental stress may correlate with an increased risk of ADHD development, potentially mediated by the increase of sensory and emotional dysregulation; (ii) This increased sensory and emotional dysregulation give rise to the ‘classical’ ADHD symptoms of inattention, impulsivity, and hyperactivity. These symptoms may be diagnosed through (iii) objective, physiological means, and finally (iv) mitigated with MPH application, and, following further research, possibly also by treatments aimed at these dysregulation processes. To properly further aid ADHD individuals, further research is required to better define causes, symptoms and preventative techniques. Further research is also needed to determine the role of early-life stress effects on the development of ADHD, particularly as there is limited clinical evidence for the causative role of early-life stress on ADHD development. This creates a limitation within our review paper, as the lack of research limits the potential number of references. A deeper investigation into the additional suggested symptoms of ADHD (emotional and sensory dysregulation) beyond the current core symptoms of impulsivity, hyperactivity, and inattention, bares an important potential in improving both ADHD diagnosis (and differential diagnosis) and treatment.

In sum, better ADHD treatment strategies may be achieved by the inclusion of further research on (i) developmental stress on ADHD development, (ii) emotional and sensory dysregulation as potential ADHD additional symptoms, and finally (iii) the effects MPH on emotional and sensory dysregulation in ADHD populations. This new and comprehensive prism could lead to new tools for diagnosing and treating individuals with ADHD.

## Author contributions

AG surveyed the literature, drafted the manuscript, and co-wrote the conclusion. AA conceptualized the manuscript, co-wrote the conclusion, and edited previous versions into the final one. Both authors contributed to the article and approved the submitted version.
